# Medicine in Its Relations with Botany

**Published:** 1874-07

**Authors:** W. T. Grant

**Affiliations:** Georgia


### MEDICINE IN ITS RELATIONS WITH BOTANY.

BY W. T. GRANT, M.D., OF GEORGIA.

In a recent article published in this Journal, I stated that the dichotomous analysis could be applied to medical botany. I wish now to explain the theory of such application, as it has presented itself to my mind for some years past.

I have given a good deal of time during the last fifteen years, to the practical study of botany, and may therefore claim some acquaintance with the principles of this science. Another purpose I have also in view, is to bring to the notice of the profession the great necessity of cultivating this branch of science. My study and thoughts upon the subject, lead me to the conclusion that there is an *absolute necessity* for the establishment of a chair of botany in every medical college. And it is my purpose, if circumstances permit, to discuss this point more fully, before I have done with the general subject.

The essence of the theory which I have formed in connection with the subject of medical botany, is as follows: I believe that *in every plant which possesses medical virtue, there is a physical character, or some combination of such characters, which indicates the fact. And that either the same physical characters, or others, will also indicate the* SPECIAL KIND *of medical virtue residing in the plant.* The external or physical characters of plants are thus presented in a new light, that is, they will serve to show what virtue is to be found in any plant. I may illustrate this idea as follows: Suppose it is found that whenever a plant has *white* flowers, it is always *emetic.* If such a connection should be found invariably present in all *known* plants, we should have excellent reason for expecting the same sequence among *unknown* plants. And the *white flower* would be accepted as an index showing the presence of emetic virtues. And the conclusion would be legitimate that other virtues would have other indices, to indicate their presence. And our whole object would then be to ascertain what are indices.

The variations among the physical characters of plants are nature's landmarks. And the very best variation, even of form or color, has a purpose, and *may* possess a value not now dreamed of in our philosophy. There is a beautiful network of laws interwoven through all plant-life, and comes far more closely in contact with the rough exterior physical characters of plants than we imagine. Their physical characters are the alphabet of plant-life, or rather of vegetable therapeutics, and if combined into words and sentences, will constitute the language in which nature has written these laws. And if we can but learn to read it, we have before us in every plant we meet, a manuscript, wherein we may read clearly and distinctly some of nature's profoundest secrets. And thus we may become possessed of the key which will open to view the entire field of therapeutics, and the practice of medicine would become simply the application of a few plain rules, and we could foresee the course to pursue in any given case, as surely as we now do in working out a simple sum under the rule of three.

If there be any truth in these thoughts, the object we should have in view, would be to determine the different indices, as above mentioned. Or, in other words, ascertain the connection between the physical characters of plants and their therapeutic virtue. To do this it will be necessary to go over the entire field of descriptive botany. And I doubt whether descriptive botany is at present sufficiently minute and exhaustive to answer our purpose. Nor would the classifications now used in botany afford any assistance. It is true they are all founded on the natural characters of plants; still they are artificial, and cannot be used for any other purpose than to point out a few of the external or physical *resemblances* existing among plants. It therefore follows that we can never with safety draw the conclusion that because a certain plant exhibits certain physiological effects, that therefore another certain plant will exhibit similar or analogous effects, simply because we find the two standing in close juxtaposition in the present classifications. This is not even true of the different species of the same genus, as is shown in solanum. Much less is it true of the different genera of the same natural order. It will also be evident at once, that present botanical classifications are useless for our purpose, by the consideration that one of the results of our study, will be to bring together, plants widely seperated in them.

To determine the different indices will require in the investigator an accurate knowledge of practical botany, together with a thorough knowledge of physiology and therapeutics. A botanist, however well informed he may be in botany, is not as well prepared to investigate this subject as he should be. It thus appears that the study of botany should be combined with that of medicine, before we can have properly prepared investigators in the field. Consequently there is an absolute necessity for a chair of botany in every medical college.

If we were in the woods and find a strange plant which attracts our attention, how shall we know whether or not it is poisonous? Or whether it is a valuable or worthless plant? I have not the least doubt that there is some arrangement of some of its parts, which would tell us, if we could only interpret them correctly. The special plan or color of the flower; the arrangement of the leaves; the number or form of the stamens or petals or pistils, or indeed any one of the infinite variations which any plant may exhibit, would be to us the desired index, if we could but read it aright.

This doctrine that *there is always an invariable connection between the physical characters of plants and their physiological effects,* has never before been distinctly announced, but it has had a latent existence, as is seen when we read in some of the older authors on botany that a plant may be known to be poisonous by its having *five stamens.* And it is now stated, that the poisonous species of the gevas rhus (common sumach), may be distinguished by their flowers being always axillary.

In studying this subject, another thought has suggested itself to me, which may be serviceable in its elucidation. It has often occurred to me that there is an intimate connection between the different parts of a plant. And for convenience of explanation, I shall call it the doctrine of complements. This means that every part is complementary to some other part of the same plant, and that between such complementary parts, there is a constant relation. So that whenever any one part of a plant is indefinitely developed, or otherwise changed, its complementary part undergoes a corresponding change. It cannot well be doubted that there is some such relationship or *sympathy* (to give more accurate expression to the connection) between the parts of the *flower,* especially the gynæsium and andræsium, by which the glutinous surface and penetrability of tissue of the stigma, have attained perfection just at the moment when the anther, having perfected its pollen, bursts and scatters the pollen grains upon the stigma. Every one sees this in corn every year. How is it that the silk and the tassel both mature, and the one is ready for the other just at the right time, although they are on different parts of the plant? The maturation of the silk and the tassel are distinct operations, and are performed by distinct parts of the plant, as it is in all plants; both are essential, and both are equally necessary to the perfection of the seed; so that there can by no possibility be any *chance* or *accident* in their operations. And thus there is no escape from the conclusion that there is some mutual reaction or sympathy between the male and female organs, which enables the one to perform its functions in union with the other. And if this be true, an extension of the same principle to all other parts of the plant will constitute the relationship which I have called the doctrine of complements.

I now come to the consideration of the special object of this monograph, which is the application of the Dichotomous analysis. I do not suppose for a moment that the Dichotomous analysis will or can assist in the developement of the subject I have tried to present intelligibly above. But it will constitute the medium by which all the above results, attained by prolonged investigation, is to be presented in one view to the profession. When the indices shall all have been ascertained, such an analysis can be constructed, which will lead through a series of Dichotomes to the medical virtue of any plant, just as we can now ascertain the name of a disease by the analysis of diseases. And if such an analysis is ever constructed, any one can go into the woods with it, and at sight determine the medical value of any plant, even though it should never before have been seen. Such is certainly a most desirable consumation, and must enlist the sympathy, if not the active coöperation of every one.

I will illustrate the Dichotomous analysis which is proposed to be constructed on three principles, by using the following slightly altered analysis, and substituting therapeutic virtues for the names. I use the genus houstonia, and the following is the analysis of the species as given in Wood's botany:

| | | |
|---|---|---|
| 1. | Corolla Salverform-glabrous, Peduncles 1 flowered | 2 |
| 1. | " Funnelform " many " Cymose | 3 |
| 2. | Flowers terminal | H. Cœrulia. |
| 2. | " axillary | H. Minima. |
| 3. | Leaves Lance-Ovate | H. Purpurea. |
| 3. | " " -Linear | H. Longifolia. |

I translate this by the following substitutions: Substituting the generic name "stimulants" for "houstonia," the result is the following analysis :

1. Corolla Salverform-glabrous, Peducles 1 flowered.....(Nervons Stimulants)..2
1. " Funnelform " many " Cymose.(Arterial " )..3
2. Flowers terminal..............................Affecting only the Brain.
2. " axillary.......................Affecting the Exitory-Secretory Nerves.
3. Leaves Lance-Ovate..............Affecting the Heart and General Circulation.
3. " " -Linear ................Affecting the Capillary Circulation only.

This example illustrates the fact that if the plan of the flower and the leaves as given in the first analysis above, were demonstrated to be the indices to point to the fact that those plants acted as stimulants, and each one had a special effect different from the others as it differed in those special physical characters, then it follows that the second of the above analysis will be a correct and exact expression of these facts.

I am not prepared to attempt to construct the Dichotomous analysis which will by the object and aim of all the labor that may be bestowed upon this subject. In the course of my studies of this subject, I have developed many crude ideas, which by further study may assume definite form and shape, and develope into real truths. I will only suggest the following as the first general Dichotome, but without insisting that it can be maintained :

1. Plants having some one or more parts indefinitely developed, and which have not Umbellate flowers......................Are Safe.
1. Plants having no one or more parts indefinitely developed, or which have not Umbellate flowers..........................Are Suspicious.

Under the first division of this Dichotome, an exception is made of all *umbelliflorate* plants. In using the word *umbelliflorate*, I mean exactly what is conveyed by the word. Not the umbellifers alone, but every plant which has an umbellate infloresence. This will embrace the natural order asclepiadaceæ, together with a large number of individual plants belonging to other orders. I am strongly disposed to consider an umbellate infloresence as certain evidence of dangerous properties in any plant. If this should prove true, we have in it an index available in the proposed analysis.

I do not know whether I have or have not succeeded in conveying a very accurate idea of the theory which I have formed on the subject of medical botany. If I have, many confirmatory thoughts will occur to every mind after a little consideration of the subject, which it is impossible for me now to mention.

It is true that all the thoughts above suggested are theoretical. But all great results have arisen from the search for evidence to support some preconceived theory. And even though we may never be able to construct the Dichotomous analysis which I have suggested, yet medicine may be incalculably benefitted by the collateral valves developed in the investigation. Astrology was cultivated and trusted for centuries, as a means of foretelling future events. And the science of astronomy is one of its unforeseen and unexpected results. The investigations and experiments of alchemy were all directed to the discovery of the philosophers' stone, and chemistry is a most invaluable collateral result of those studies. Neither in astrology, nor in alchemy, was the direct purpose ever accomplished, yet those pursuits were not without profit, as astronomy and chemistry, their bastard offspring, abundantly prove. And as they have rendered infinite service to the world, we thus have presumptive reason to expect that laborious researches in the new fields which the theory I offer presents, will not be altogether barren of results. If the main purpose be not accomplished, yet many valuable facts will be developed, and medicine greatly enriched by the study of a science, heretofore ostracised by the profession; and which I believe is capable of indefinitely extending the bounds of our knowledge in therapeutical medicine.

It is an unfortunate fact that while every other branch and department of human knowledge and industry has made wonderful progress during the last quarter of a century, medicine alone lags in the rear, and the physician of to-day, by excluding one or two improvements, may use as a reference or as a text-book, almost any author of twenty-five years ago. This is especially true of therapeutics. In every other human pursuit the author of one year is now superannuated by the improvements of the next. But this is not true of the medical sciences.

